# Minimal changes of serum creatinine in the early postoperative period predict prognosis in patients after cardiac surgery

**DOI:** 10.1186/2197-425X-3-S1-A636

**Published:** 2015-10-01

**Authors:** MH Bernardi, R Ristl, M Mouhieddine, M Hiesmayr, A Lassnigg

**Affiliations:** Cardiothoracic and Vascular Anaesthesia & Intensive Care, Medical University of Vienna, Vienna, Austria; Center for Medical Statistics, Informatics and Intelligent Systems, Medical University of Vienna, Vienna, Austria

## Introduction

Preoperative renal insufficiency is an important predictor of mortality after cardiac surgery and the association between small serum creatinine (SCr) changes within 48 hours after cardiac surgery and mortality has been demonstrated. ([1]) Further it has been shown recently that a preoperative elevated SCr is a predictor for worse outcome after cardiac surgery too. ([2])

## Objectives

The aim of the present investigation was the association between small SCr changes (ΔCrea) early after surgery on 30-day mortality in patients below and above the SCr cut-off of 1.3 mg.dL^-1^ where mortality increases.

## Methods

Elective adult cardiac surgical patients between 1997 and 2001 at the Medical University of Vienna were included. The cohort was split into two groups: Patients with an elevated SCr >1.3 mg.dL^-1^ and ≤1.3 mg.dL^-1^. Within 120 minutes after end of surgery, the ΔCrea between the first measured SCr and the baseline SCr value was calculated for each patient. Mortality rates were calculated stepwise in 0.1 mg.dL^-1^ intervals of ΔCrea.

## Results

A total of 3549 patients (1221 women) with a mean age of 64.5 years (range, 18 to 92) were investigated, 624 patients had an elevated SCr >1.3 mg.dL^-1^. Within 30 days 5% (n=179) died. Mortality in patients with elevated SCr and a negative ΔCrea or equal 0 was 9% (n=573), while mortality increases in patients with an increasing ΔCrea to 31% (n=51).

**Figure 1:**
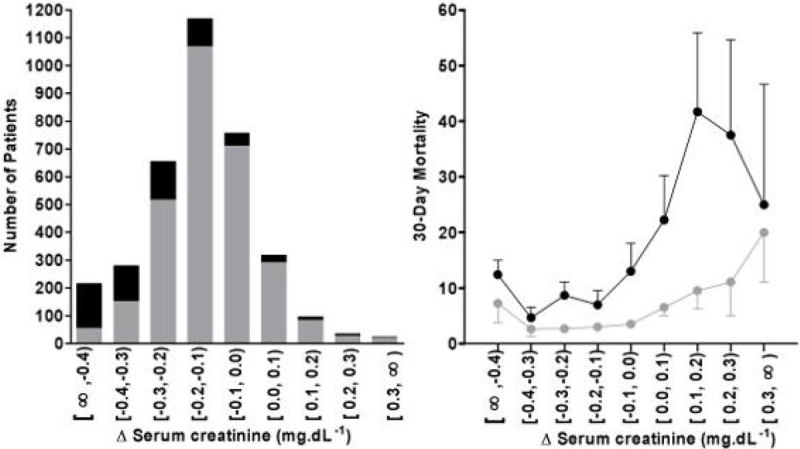
Figure 1

An increase in mortality (12%) was found in the group with the most pronounced fall [∞,-0.4). The relation of mortality to ΔCrea is shown in figure 1 (SCr >1.3 mg.dL^-1^marked in black and ≤1.3 mg.dL^-1^ marked in grey).

## Conclusions

Hemodilution occurs in patients operated on cardiopulmonary bypass (CPB). A decrease in SCr is the reaction to fluid supply and blood loss especially when preoperative SCr is elevated. Our findings suggest that in these patients with an elevated SCr, even a minimal increase after CPB accordingly to the preoperative SCr is associated with a higher rate of death and may be a marker of diffuse organ injury. Furthermore large decreases of ΔCrea directly after cardiac surgery worsen outcome in this patient group drastically. A renoprotective postoperative course is recommended.
